# Biomechanical Comparison and Three-Dimensional Analysis of Cement Distribution Patterns for Different Pedicle Screw Designs

**DOI:** 10.1155/2022/8293524

**Published:** 2022-10-18

**Authors:** Sahyun Sung, Ji-Won Kwon, Tae Hyun Park, Soo-Bin Lee, Seong-Hwan Moon, Byung Ho Lee

**Affiliations:** ^1^Department of Orthopedic Surgery, Yonsei University College of Medicine, 03722 Seoul, Republic of Korea; ^2^Department of Orthopedic Surgery, Ewha Womans University College of Medicine, 07804 Seoul, Republic of Korea; ^3^School of Biomedical Engineering, Inje University, 50834 Gyeongnam, Republic of Korea; ^4^Department of Orthopedic Surgery, Catholic Kwandong University International Saint Mary's Hospital, 22711 Incheon, Republic of Korea

## Abstract

The purpose of this study to explore strategies for reducing cement leakage during cement-augmented pedicle screw fixation, we compared the cement distribution patterns and biomechanical strengths of different types of cement-augmented fenestrated screws and traditional cement-augmented techniques. We compared five screw groups in this study: (1) Cannulated screws (Cann); (2) distal one-hole screws (D1); (3) distal two-hole screws (D2); (4) middle two-hole screws (M2); and (5) traditional screws with a traditional cement injection technique (Trad). The screws were inserted into cancellous bone blocks using a controlled, adequate cement injection pressure (1.6–2.0 kg), and an appropriate cement viscosity. Center to screw tip distance, three-dimensional distribution, and pull-out strength for cement were compared between groups. The average distance between the cement center and the screw tip was highest in the M2 group, suggesting a higher risk of cement leakage into the spinal canal. The Trad group had the highest migration distance in the *z*-axis, also reflecting a higher risk of leakage into the spinal canal. The D1 group had the highest pull-out strength (253 ± 48.82 N and 797 ± 58.31 N) in bone blocks representing different degrees of osteoporosis, and the D2 group had the second highest pull-out strength in the severe osteoporosis model. Overall, D1 screws appeared to be the best option for optimizing biomechanical function and minimizing the risk of cement leakage into the spinal canal in patients with osteoporotic bone undergoing spinal surgery.

## 1. Introduction

The number of older adult patients with osteoporosis undergoing surgery for degenerative spine conditions has increased with longevity. [1–6] In patients undergoing spinal fusion surgery with pedicle screws, proper bone quality for maintaining the fixation devices is essential to prevent screw pull-out.

Polymethylmethacrylate cement augmentation and screw fixation are commonly used to treat osteoporotic thoracolumbar spines. [7, 8] Numerous methods have been introduced to improve bone quality, [9] including cement-augmented screw fixation. [10–12] Cement-augmented techniques, however, have potential problems, including cement leakage into the spinal canal and neurologic complications. [13, 14] Nevertheless, few studies have evaluated the optimal design of cement-augmented screws to reduce cement leakage-related complications. [15]

Therefore, we modeled four different cement-augmented fenestrated screws and compared the cement distribution patterns and biomechanical strengths of these screws with those of traditional cement-augmented techniques.

## 2. Materials and Methods

### 2.1. Synthetic Bone

Commercial synthetic bone (Sawbone; Pacific Research, Inc., Vashon Island, WA, USA) was used as a model for cancellous vertebral body bone because of its homogenous and uniform structural properties. To simulate clinical conditions, three different types of synthetic bone were used: (1) open cell rigid form, grade 7.5 pounds per cubic foot (pcf), representing osteoporotic bone, (2) open cell rigid form, grade 15 pcf, representing osteoporotic bone treated with medication or osteopenic bone, and (3) solid form, grade 15 pcf, representing normal bone. The density of cancellous bone model for each disease was determined by referring to “Standard Specification for Rigid Polyurethane Foam for Use as a Standard Material for Testing Orthopaedic Devices and Instruments.” [16, 17] Each synthetic bone part was cut into rectangular blocks at dimensions of 40 mm × 90 mm × 65 mm.

### 2.2. Pedicle Screw System

**Figure 1:**
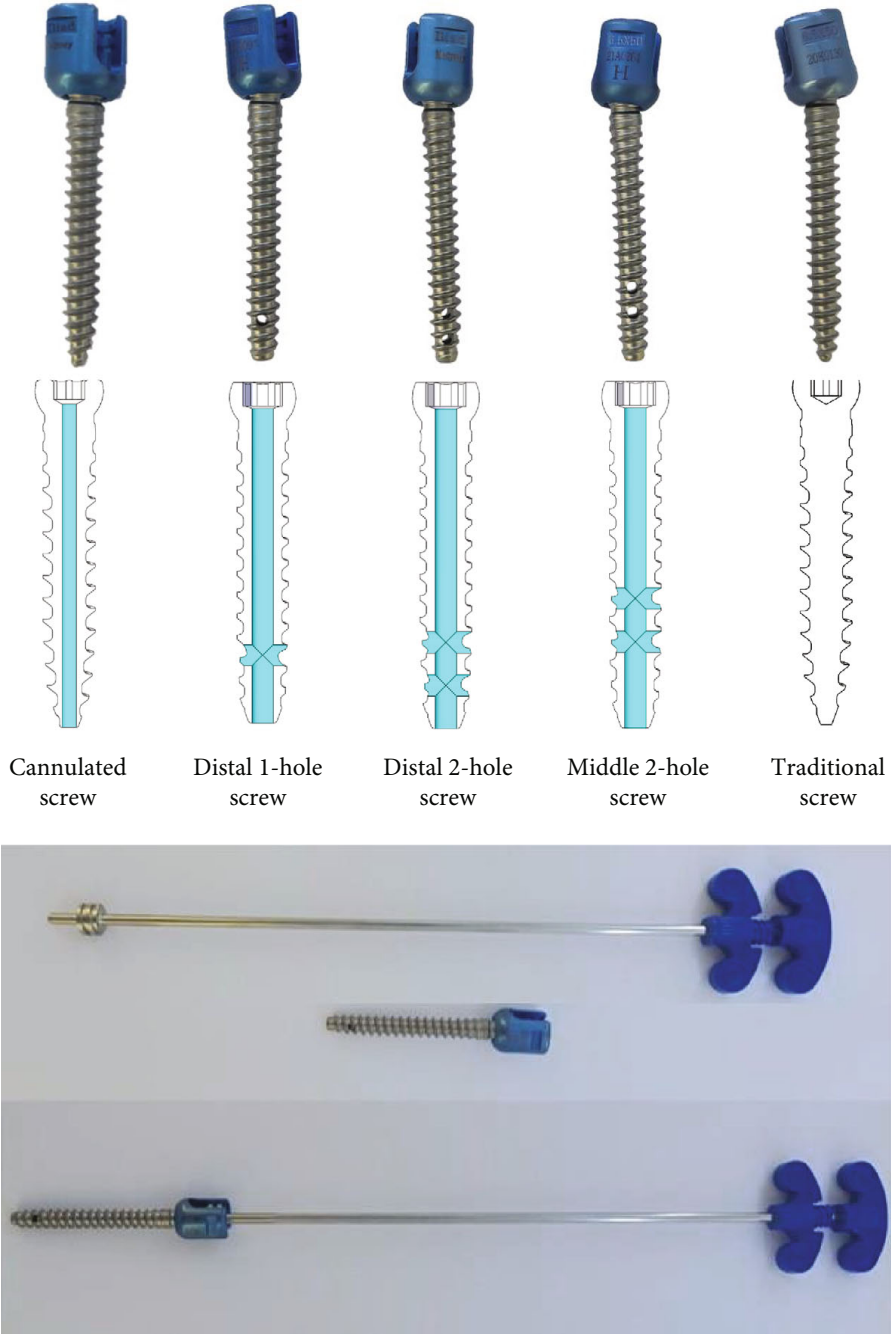
Cement screw design and cement injector in the Iliad™ pedicle screw system.

The Iliad™ pedicle screw system (Medyssey Co., Ltd., Jecheon, Korea) was used. Each screw had a length of 50 mm and a diameter of 6.5 mm. Five types of screws were used: (1) cannulated cement screws (cannule-only screws used in the percutaneous pedicle screw system) (Cann); (2) distal one-hole screws (D1); (3) distal two-hole screws (D2); (4) middle two-hole screws (M2); and (5) traditional screws with a traditional cement injection technique (Trad) (Figures 1).

### 2.3. Preparation of the Cement Injection Model

Commercially available cement (Exolent spine cement, Elmdown, England) was mixed in a liquid-to-powder ratio of 20 mL:36 g, which is similar to the ratio used clinically. Three mL was chosen as the volume of injected cement based on previous literature. [18, 19]

**Figure 2:**
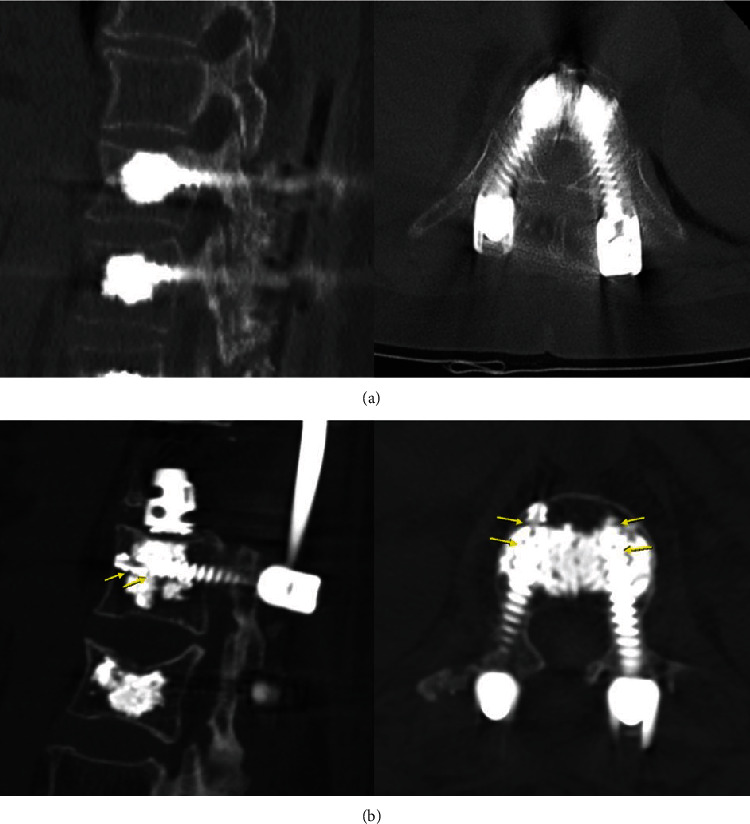
Cement distribution pattern of traditional cement method and pressurized method confirmed in clinical computed tomography (The guide arrow indicate the side hole of fenestrated screw.).

Cement was injected using the pressurized method for the Cann, D1, D2, and M2 screw groups and using the traditional augmented method for the Trad screw group. In the pressurized method, a 5.5 mm diameter tapper was used on a precut synthetic bone block to form a 40 mm vertical channel, after which the screw was inserted leaving a 5 mm screw neck margin. Cement (3 mL) was then injected into the screw using an MVP™ injection kit (Medyssey Co. Ltd.), with the injection pressure controlled between 1.6 and 2.0 kg. (Figure 1) In clinical cases, when cement is injected using traditional augmented method the cement is distributed longitudinally, whereas when the fenestrated screw is used, the cement forms a ball shape around the hole (Figure 2).

### 2.4. Distribution of Injected Cement

To determine the location and distribution of injected cement, micro-computed tomography (Quantum FX *μ*CT; Perkin Elmer Co. Ltd., USA) was performed using 148 *μ*m thick slices. Images were saved in the Digital Imaging and Communications in Medicine format and then analyzed using MIMICS software, version 21 (Materialise Co. Ltd., Belgium). This software was developed for medical image segmentation and 3D model reconstruction. Hounsfield units were adjusted for each structure in the CT system, using 3,000–6,500 HUs for the cement. The geometry of the cement was then measured using 3-matic software (Materialise Co. Ltd.).

To determine the distribution of injected cement, we measured *x*-, *y*-, and *z*-axis dimensions. The direction of the hole was designated as the *x*-axis to examine the effect of cement distribution exiting through the fenestrated hole. The center of the injected cement was calculated using the analytical fitting function, and the distance was measured between the center and the screw tip.

### 2.5. Pull-Out Strength (Mechanical Test)

The screw pull-out test was performed using an MTS 858 Bionix test machine (MTS; Minneapolis, MN, USA). The applied loads were measured by an axial load cell (661.18H-02, 3.3 kN maximum axial load); the displacement transducer was an MTS LVDT transducer (370.02 A/T, 150 mm range). The test block, with an inserted screw, was placed under a specially designed fixture system to axially constrain the block to the test machine; this is standard methodology for pull-out tests, including those described by ASTM F543. [20]

After a specimen was mounted, the pull-out load was applied under displacement control at an actuator rate of 2 mm/min until screw pull-out was observed. Values measured by the load cell and transducer sensor were plotted in a load-displacement curve for each test. The pull-out force was defined as the first peak force measured during axial ramp loading. [21]

### 2.6. Statistical Analysis

Each screw design was measured by preparing six grade 7.5 specimens and six grade 15 specimens, respectively. Data are presented as a mean ± standard deviation, unless otherwise indicated. Since screw design groups did not satisfy normality, the omnibus test was performed using Kruskal-Wallis test, and then Mann–Whitney test was performed for each group as a post hoc test. Differences were considered significant at *P* < 0.05.

## 3. Results and Discussion

### 3.1. Results

#### 3.1.1. Distance between the Cement Center and Screw Tip

**Figure 3:**
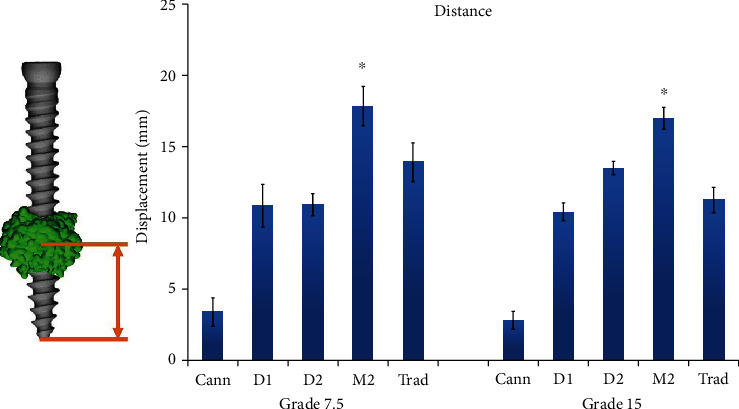
Distance between the center of the spherical cement ball and the distal screw tip.

Figure 3 and Table 1 depict the distances between the center of the spherical cement ball and the distal tip of the screw for each experimental group. Cement distribution was more consistent in grade 15 bone specimens than in grade 7.5 specimens, with a smaller standard deviation.

In the grade 7.5 bone blocks, the distance between the center of the cement ball and the distal screw tip was highest in the M2 group (17.87 ± 1.38 mm) (Figure 3). Distances in the other groups, in descending order, were as follows: Trad, 13.92 ± 1.36 mm; D2, 10.94 ± 0.76 mm; D1, 10.85 ± 1.51 mm; and Cann, 3.38 ± 0.99 mm. In the grade 15 bone blocks, the distance was also highest in the M2 group (17.00 ± 0.77 mm). Distances in the other groups, in descending order, were as follows: D2, 13.51 ± 0.46 mm; Trad, 11.28 ± 0.90 mm; D1, 10.43 ± 0.62 mm; and Cann, 2.81 ± 0.63 mm. In both grade 7.5 and grade 15 bone, distances in the M2 group were significantly higher than those in all other groups (Mann–Whitney *U* test, *P* < 0.05).

**Figure 4:**
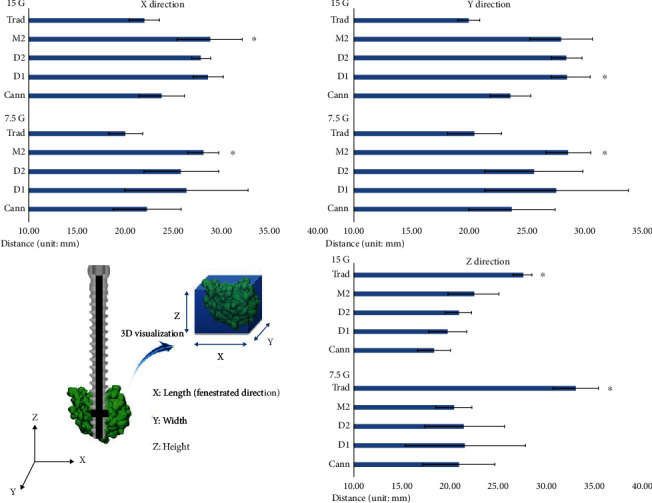
Three-dimensional analyses of cement distribution.

In the *x*-axis, the M2 group showed the highest distance in the grade 7.5 bone block (28.24 ± 1.58 mm; Mann–Whitney *U* test, *P* < 0.05) (Figure 4). Distances in the other groups, in descending order, were as follows: D1, 26.47 ± 6.40 mm, D2, 25.49 ± 3.92 mm, Cann, 22.34 ± 3.55 mm, and Trad, 20.09 ± 1.78 mm. In the grade 15 bone block, the distance was also highest in the M2 group (28.89 ± 3.37 mm). Distances in the other groups, in descending order, were as follows: D1, 28.69 ± 1.56 mm; D2, 27.97 ± 0.96 mm; Cann, 23.87 ± 2.37 mm; and Trad, 22.07 ± 1.51 mm.

In the *y*-axis, the M2 group showed the highest distance in the grade 7.5 bone block (28.54 ± 1.90 mm) (Figure 4). Distances in the other groups, in descending order, were as follows: D1, 27.50 ± 6.20 mm; D2, 25.56 ± 4.25 mm; Cann, 23.65 ± 3.74 mm; and Trad, 20.43 ± 2.338 mm. In the grade 15 bone block, the distance was highest in the D1 group (28.44 ± 1.98 mm). Distances in the other groups, in descending order, were as follows: D2, 28.39 ± 1.32 mm; M2, 27.94 ± 2.64 mm; Cann, 23.55 ± 1.73 mm; and Trad, 19.91 ± 0.93 mm.

In the *z*-axis, the Trad group showed the highest distance in grade 7.5 bone blocks (32.95 ± 9.13 mm) (Figure 4). Distances in the other groups, in descending order, were as follows: D1, 21.44 ± 4.47 mm; D2, 21.31 ± 3.49 mm; Cann, 20.79 ± 1.74 mm; and M2, 20.30 ± 1.18 mm. In the grade 15 bone block, the distance was highest in the Trad group (27.49 ± 2.62 mm). Distances in the other groups, in descending order, were as follows: M2, 22.35 ± 3.38 mm; D2, 20.83 ± 0.82 mm; D1, 19.63 ± 1.19 mm; and Cann, 18.25 ± 1.80 mm.

#### 3.1.2. Screw Pull-Out Strength

**Figure 5:**
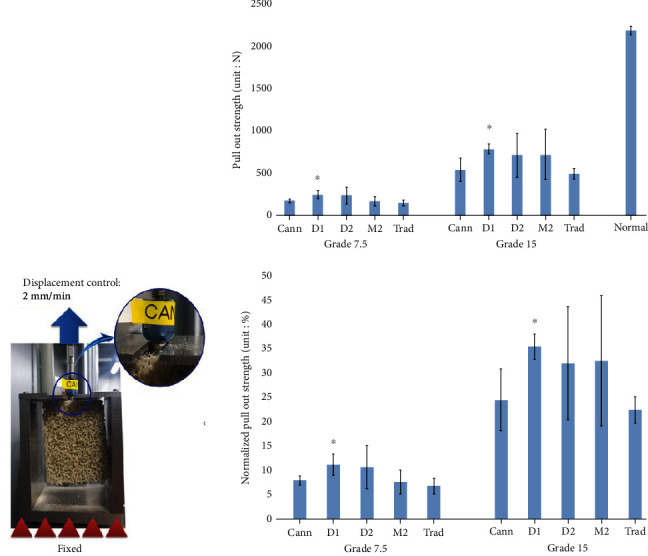
Comparison of Pull-out strength and normalized pull-out strength.

Compared to the pull-out strength of conventional screws in the normal strength bone block, all cemented screws exhibited lower pull-out strength in both open cell grade 7.5 and 15 bone blocks (Figure 5). Among the cemented screws, the D1 group had the highest pull-out strength in both bone blocks (253 ± 48.82 N in grade 7.5 bone; 797 ± 58.31 N in grade 15 bone) (Mann–Whitney *U* test, *P* < 0.05). In the grade 7.5 bone block, pull-out strengths in the other groups, in descending order, were as follows: D2, 240 ± 99.59 N; Cann, 179 ± 20.88 N; M2, 171 ± 55.53 N; and Trad, 153 ± 36.49 mm. In the grade 15 bone block, pull-out strengths in the other groups, in descending order, were as follows: M2, 733 ± 301.30 N; D2, 722 ± 260.78 N; Cann, 551 ± 143.61 N; and Trad, 504 ± 62.34 mm.

We also assessed normalized pull-out strength, defined as the ratio of the pull-out strength of the experimental groups to that of noncemented traditional screws in normal strength bone. Normalized pull-out strength was significantly higher in grade 15 bone than in grade 7.5 bone (Figure 5). In the grade 7.5 bone block, normalized strength was highest in the D1 and D2 groups (11.0 ± 2.20% and 11.0 ± 4.49%), followed by the Cann, M2, and Trad groups (8.0 ± 0.94%, 8.0 ± 2.50%, and 7.0 ± 1.64%, respectively). In grade 15 bone, normalized strength was highest in the D1 group (36.0 ± 2.63%), followed by the M2, D2, Cann, and Trad groups (33.0 ± 113.58%, 33.0 ± 11.76%, 25.0 ± 6.47% and 23.0 ± 2.81%, respectively).

The results and discussion may be presented separately, or in one combined section, and may optionally be divided into headed subsections.

## 4. Discussion

In patients with osteoporosis, cement-augmented pedicle screws are a good option to enhance bone screw fixation until bony fusion is achieved. [7, 22, 23] Because of concerns for cement leakage, other options such as expandable screws and polymethylmethacrylate substitutes have been explored. [19] However, only a few studies have reported on the use of these alternatives, and their use has been limited. [19] The use of cement-augmented pedicle screws is easy and feasible, although biomechanical failure can occur if an inadequate amount of bone cement is used or cemented screws are misplaced. [24] In a series of studies, loosening rate was significantly lower in cement-augmented pedicle screws (4.3%) than noncemented screws (62.8%). [25] There have been recent reports on the clinical efficacy of cement augmentation using fenestrated screws. [26–29] Studies have reported the safety and effectiveness of fenestrated pedicle screws in not only osteoporosis but in other conditions, which can induce vertebral instability such as tumor. [30]

We quantified the distribution of injected cement to predict the likelihood of cement leakage in real-world clinical settings. Although fluoroscopy was not utilized during cement injection in this study, a consistent sphere-shaped cement mass, which would be associated with a minimal risk of cement leakage, was achieved in all experimental groups, except for the Trad screw model. The *z*-axis distribution of the cement mass, which is most important in preventing cement leakage into the spinal canal, was highest in the M2 and Trad groups. It is also important to note that the distribution of cement was greater in the grade 7.5 bone model, representing more severe osteoporosis, than in the grade 15 bone model. Although the viscosity and injection pressure of cement were controlled in the Trad group, as they were in the other groups, the cement was located along the screw insertion tract, which could result in cement leakage into the spinal canal in clinical settings. In contrast to Trad cement screw insertion, other models demonstrated a consistent pattern of cement distribution, depending on the position and number of cement holes.

Another important finding in this study was that the traditional cement injection technique was biomechanically weaker and thus likely to be less effective than the other cemented screw techniques. The pull-out strength of the traditional screws was similar to only that of the Cann screws in both grade 7.5 and grade 15 bone. The superior strength achieved using the D1, D2, and M2 screws may be attributed to a direct cement core connecting the cement ball surrounding the screw with the cement remaining inside the screw shaft. Thus, the greater pull-out strength may be explained by the screw design. We observed no breakage or separation of this core in any of our experiments. D1 screw had the highest pull-out strength. This could be because since the distance from the screw tip to the center of the cement mass was lowest among the fenestrated screws, there are more amount of cancellous bone between the cement mass and the pedicle surface, which prevents screw pull-out.

There were some limitations to our biomechanical experiments. First, the applied physiologic loading condition may not exactly represent the forces encountered in clinical practice. However, pull-out tests have been used extensively to measure biomechanical holding power and stability. [31] Although pure pull-out is not frequently seen in clinical situations, pull-out testing is still considered a good predictor of pedicle screw fixation strength. [32] Most screw failures in osteoporotic patients occur due to screw dislodgement from the most cephalad or caudal end of screw-rod constructs. [15, 33, 34] Second, synthetic bone differs from real pedicle-body bone because it lacks a cortical layer. Thus, the biomechanical environment in the current study does not completely represent actual physiologic situations; however, our experiments still provide considerable information for reference because the augmented cement mass should be located solely within the cancellous portion of bone and not be in contact with the cortical portion of the pedicle. However, many biomechanical studies related to cement/calcium phosphate-augmented screw systems were based on sawbone models. [19, 21, 35] Additionally, if the cortical portion is contacted, posterior migration of cement-augmented screws or cement leakage into the spinal canal can occur.

Fortunately, all screw designs evaluated in this study have been approved for clinical use by the Korean Ministry of Food and Drug Safety, so we can use our results to select screws, which is least likely to produce cement leakage for future clinical investigations. We are currently undergoing a randomized controlled study to confirm our findings in this study in clinical setting. [36]

## 5. Conclusions

In conclusion, this is the first study to demonstrate the safest design of cementable fenestrated screws via a controlled, adequate injection pressure. Overall, distal one-hole fenestrated screws appeared to the best option and distal two-hole fenestrated screws appeared to be the second-best option for clinical usage in patients with osteoporotic bone undergoing spinal surgery.

## Figures and Tables

**Table 1:** Average distance between cement ball center and screw tip by screw designs.

Distance (mm)	Cann		D1		D2		M2		Trad	
	M	SD	M	SD	M	SD	M	SD	M	SD
7.5G (osteoporosis model)	3.38	0.99	10.85	1.51	10.94	0.76	17.87	1.38	13.92	1.36
15G (osteopenia model)	2.81	0.63	10.43	0.76	13.51	0.46	17.00	0.77	11.28	0.90

Abbreviation: Cann: cannulated screw; D1: distal one-hole screw; D2: distal two-hole screw; M2: middle two-hole screw; Trad: traditional screw; M: mean, SD: standard deviation. ^∗^Statistically significant results were shown when compared with each other group through Kruskal-Wallis test.

## Data Availability

The data will be available upon reasonable request.
